# Evaluation of a ‘Research Methods’ Training Course for Novice Lived Experience Researchers

**DOI:** 10.1111/hex.70362

**Published:** 2025-08-28

**Authors:** Andrew C. Grundy, Anam Bhutta, Ashgan Mahyoub, Rebecca Jenkins, Sadia Mir, Jahanara Miah, Gail Faragher, Karina Lovell

**Affiliations:** ^1^ Division of Nursing Midwifery and Social Work, School of Health Sciences, Faculty of Biology, Medicine and Health, University of Manchester; National Institute of Health and Care Research, Applied Research Collaboration—Greater Manchester (NIHR ARC‐GM) Manchester UK; ^2^ Mental Health Research for Innovation Centre (M‐RIC) Mersey Care NHS Foundation Trust and the University of Liverpool Liverpool UK

## Abstract

**Background:**

The training of lived experience researchers (LERs) for involvement in research design and conduct is a key principle to its success. However, little is known about what research methods training is acceptable and beneficial to novice LERs.

**Methods:**

A training evaluation using a slightly modified version of the *Training Acceptability Rating Scale* (TARS), and a concluding stakeholder engagement workshop. Responses to the quantitative items were summarised using descriptive statistics, and qualitative responses were coded using content analysis.

**Results/Findings:**

The trainees rated the overall training favourably (median overall TARS = 54/63; median acceptability = 31/36; median perceived impact = 23/27), but there was slight variation between sessions. There were six qualitative themes: valued learning format; valuing research knowledge; valued the centring of lived experience; gaps in training provision; more lived experience research focussed; and consider further support of LERs.

**Conclusions:**

The training was found to be acceptable and beneficial, with trainees particularly valuing lived experience facilitation, case studies and tailored content. Trainees suggested the training could be improved by addressing theoretical, existential and skills gaps, and by making it more lived experience research focussed throughout.

**Patient or Public Contribution:**

This training evaluation and engagement workshop project was developed and overseen by a long‐term service user with lived experience of mental distress. The workshop topic guide was co‐designed with four people with lived experience, who were also involved in data analysis and co‐constructed the findings. Finally, this paper concludes with a commentary on this study provided by a trainee with lived experience.

## 1 Background

In the United Kingdom, a high level of what is often termed ‘Patient and Public Involvement’ (PPI) in research is strongly encouraged by research funders [1, 2]. Involvement can take the form of consultation, contribution or collaboration [3], with the involvement of lived experience researchers (LERs) seen as a high form of involvement practice. Here, ‘lived experience’ refers to knowledge, understanding and expertise gained through first hand, personal experience of a phenomenon; it refers to an experience (such as a mental health condition) as truly lived through [4]. LERs give relevant and authentic testimony to their lived experience of mental distress and related service use (or of closely supporting a family member), reflexively applying their experiential knowledge to the design and conduct of research [5]. Importantly, LERs are not merely project advisors (as per some PPI roles), but are co‐researchers on the research team [6], and they can thus be embedded in research organisations [7].

The training of PPI members for involvement in research in National Health Service (NHS) settings has long been identified as a key principle to its success [8]. Training in research has been argued as needed to ‘demystify research’ and as a vital step to bridge the ‘language gap’ between scientists and PPI members [9]. By gaining research knowledge and skills, it has been shown that training can lead to *genuine* research collaboration [10], help reduce power imbalances and work towards parity of status of LERs as co‐researchers [6]. However, some researchers and PPI contributors express a reluctance to train PPI members, arguing that it might compromise their authenticity [11].

Training for PPI contributors that has been evaluated has tended to narrowly focus on enhancing soft skills [12, 13], or on qualitative interviewing and analysis alone (e.g., [14]), leaving LERs excluded from influencing other important areas of research (e.g., reviewing the literature, analysing numerical datasets, economic evaluation, trial designs and psychometrics). Previous evaluations have suggested that research training should adopt a strengths‐based approach, acknowledging and building on PPI members' experiential expertise [15], and that it should include content delivered by LERs and sharing their experiences as LERs [6].

Thus, although research methods training has long been recommended for LER roles, little is known about what mixed‐methods training and upskilling are required, or perceived to be helpful and acceptable, for LER roles from the perspective of novice LERs themselves.

## 2 Methods

### 2.1 Aim

The aims of this project were threefold: to prepare a newly formed group of PPI contributors for a research collaboration, evaluate the acceptability and perceived impact of a training package on research methods for LERs, and consider how such training could be further adapted to better meet the needs of novice LERs.

### 2.2 Research Design

We conducted an evaluation of a training package using a mixed‐methods approach. We utilised a survey research design using an established scale to gather and analyse data on trainees' experiences and satisfaction with training. We supplemented this with a concluding stakeholder engagement workshop to capture richer and more nuanced data.

### 2.3 The Training Provision

#### 2.3.1 Previous Iterations of the Training

Initially, the training was a six‐session course developed in 2010 by a team of traditional researchers and delivered from January to June 2011. The aim was to equip service users and carers with an understanding of research methods to improve their ability to understand and confidently engage with research ([16], p. 7). The training was informed by ‘traditional pedagogy’ [17] with a normalisation approach, treating the course like any other university course and the trainees like any other student. The course has now been co‐delivered to over 10 cohorts, in different settings, both nationally and internationally (e.g., [18]). In 2018, the training was further developed into a research handbook [16].

#### 2.3.2 Current Training Provision

In early 2024, two training organisers (J.M. and G.F.) commissioned A.G. and K.L. to deliver an eight‐session version of the training for their new programme of research [19]. Discussions with the programme's newly recruited PPI group informed decisions regarding the training content. A detailed outline of the sessions is provided in Table 1. Based on A.G.'s own learning from training other LERs, new lived experience‐specific elements were incorporated into the training package (summarised in Table 2).

**Table 1:** Detailed training outline.

**Introduction to lived experience in the research process** 1. Introduction to ‘research’ and to ‘involvement’ in research 2. Introduction to the research process and where involvement fits 3. Finding/defining research questions 4. Quantitative and qualitative research questions
**Systematic reviews** 1. What is a systematic review? 2. Stages in a systematic review 3. Making sense of outcomes 4. Criticisms of systematic reviews
**Qualitative methods** 1. What is qualitative research? 2. Interview topic guides 3. Ethics in qualitative research 4. Emotions in qualitative research
**Quantitative methods** 1. What are quantitative studies? 2. Types of quantitative study design 3. Quantitative data analysis methods 4. Randomised controlled studies
**Critical appraisal** 1. Recap key features of RCTs 2. What is critical appraisal? 3. Critically appraising a quantitative paper 4. Critically appraising a qualitative paper
**Collaboration** 1. Unpacking ‘patient’, ‘public’ and ‘involvement’ 2. ‘Collaboration’ in research—case study 3. LER issues in collaborative research 4. Co‐production versus faux production
**Health economics** 1. What is Health economics? 2. Example: Health policy and health inequalities 3. How do we ensure fair shares of resources? 4. How do we decide what treatments and services to provide?
**Application** 1. Applying lived experience to research 2. *[Engagement workshop/evaluation]* 3. *[End of course celebration]* 4. *[Next steps for the programme]*

**Table 2:** New LE‐specific training content.

New content	Rationale
Introduction to the concept of ‘experts by experience’ and ‘experiential knowledge’. Self‐reflection exercise to map out areas of trainees' own mental health LE (session 1)	To combat a knowledge deficit model and emphasise a strengths‐based approach; some theoretical underpinnings for their unique roles.
An emphasis on where LE fits into each stage of the research process and its potential influence (session 1)	To help see the relevance of the whole training, the benefits of LE knowledge and expertise.
Expanding the LE case study for the qualitative session (session 3)	To demonstrate how LE can inform research design and conduct.
A section on managing emotions in qualitative research for LERs (session 3)	To understand the potential challenges of researching areas you are ‘close’ to, and the importance of support.
A whole new session on involvement and the challenges of collaboration for LERs (session 6)	To understand the differences between involvement, collaboration and co‐production. To be able to challenge tokenism and faux production.
Incorporating an LE example of collaboration in research (hidden project [20])	To demonstrate how LE can inform research design and conduct; to give novice LERs an opportunity to present their work.
A brief concluding session on applying LE to new learning (first hour of session 8)	Markers of quality in LE working, applying LE.

Each training session began at 10:00 and finished at 15:00, with around a 1‐h lunch break (12:00–13:00). Typically, the morning was divided into two training slots with a short coffee break in between (10:00–11:00 and 11:10–12:00), and the afternoon was divided up similarly (13:00–14:00 and 14:10–15:00). Trainees were reimbursed for their attendance time and travel expenses.

### 2.4 Trainers

The trainers were a LER (A.G.) and three traditional academics (K.L., P.B. and W.W.). A.G. and K.L. co‐facilitated the introduction. P.B. facilitated the session on systematic reviews, K.L. on quantitative methods and critical appraisal, and W.W. on health economics. A.G. facilitated the sessions on qualitative methods, collaboration and the application of lived experience to research. One part of the collaboration session included four LERs presenting a case study (A.B., A.M., R.J. and S.M.).

### 2.5 Delivery

The training was designed to be delivered face‐to‐face, although provision was made for people to join via Teams if they were unwell or otherwise unable to travel. There was variation as to whether training slides were made available to trainees as printed handouts. Other materials/handouts were provided in some sessions, and tablet computers were provided for sourcing reviews during session 2. The training was interactive, with opportunities for small‐group work, and questions were encouraged throughout the sessions.

### 2.6 Trainees

The training organisers invited all 22 of their newly recruited ‘involvement’ members (all having relevant lived experience) to attend the training as part of their induction. They provided information about the training, reimbursement and expenses via email and verbal communication at project meetings. The trainees had no prior experience in research or academic settings. The group considered various possible descriptions of their role (e.g., ‘survivor’/‘peer’ researcher) and opted for ‘lived experience researcher’ as it was felt to helpfully foreground the key feature of the role (lived experience) whilst emphasising that this is a formal research role (in a way that Expert by Experience does not); they also approved LER as a convenient shorthand.

### 2.7 Evaluation Methods

We used a slightly modified version of the *Training Acceptability Rating Scale* (TARS; see Supplementary Material). The TARS is a self‐reported measure consisting of a training *acceptability* subsection (TARS‐1, which has demonstrated good test–retest reliability [*r* = 0.83 *p* < 0.01] and internal consistency [0.99] [21]), a *perceived impact* subsection (TARS‐2, which has not been psychometrically tested, but has repeatedly demonstrated good face and concurrent validity [22], pp. 140–141 [23]), and three open‐ended questions on the ‘most helpful’ aspects, any ‘recommended changes’ and ‘any other comments’ regarding the training.

TARS‐1 consists of six items (general acceptability, perceived effectiveness, negative side effects, appropriateness, consistency and social validity) measured on a six‐point Likert scale ranging from ‘strongly disagree’ (score 1) to ‘strongly agree’ (score 6). We modified the phrasing of questions in two respects. The intended trainees were changed from ‘participants’ to ‘service users’—so that trainees would be considering the needs of mental health service users in particular. Based on prior learning [24, 25], the phrasing of question 3 was changed from ‘The training will probably *not* result in training that leads to harm…’ (which has often caused confusion) to ‘The training will probably *not* cause harm…’ to avoid that known confusion.

TARS‐2 consists of nine items measuring perceived impacts of the training process and trainers, on a four‐point scale from ‘not at all’ (score 0) to ‘a great deal’ (score 3). No modifications were made to the phrasing of these questions.

Three open‐ended questions conclude the TARS. We modified the ‘recommended changes’ question by adding ‘particularly if you have any recommendations for better tailoring the session to the needs of service users.’ We also slightly modified the final question, ‘Please make any other comments that you would like to offer in relation to this Research Methods session for *Service Users*’. These changes again ensured that trainees were thinking specifically about any training modifications for mental health service users.

We also ran an engagement workshop to further evaluate the training. Trainees were invited to attend this on the day of the final session. The aim of the workshop was to discuss how the training could be further adapted to meet the needs of LERs.

### 2.8 Data Collection

At the end of each training session, trainees were invited to complete the TARS. At the final session, trainees were invited to complete a TARS evaluation of the whole course. No changes to the content and delivery of the training were implemented based on the evaluation, rather it was emphasised that the feedback would enhance future iterations of the training.

At the final session, trainees were invited to attend a 75‐min engagement workshop. It followed a topic guide that was co‐designed by the five LERs. The workshop was co‐facilitated by A.G. and K.L. There was a whole‐group discussion on two questions: *‘How has the training helped you apply your lived experience to research, if at all?’* and *‘What are your unanswered questions about lived experience research in particular?’* Comments were noted down by the facilitators. Attendees were then invited to split into six groups to discuss highlighted lived experience aspects of the training and answer two questions: *‘What did you think about these aspects and why?’* and *‘Is there anything else we could improve to tailor this training to people with lived experience?’* Groups noted down any comments on flip‐chart paper, with the opportunity to provide feedback to the wider group, if they wanted.

### 2.9 Ethical Considerations

Whilst this study did not require formal ethical approval, it was conducted according to ethical principles. There was a distress protocol for the training, and J.M. and G.F. were present in a support capacity. The completion of the TARS was voluntary, and it was also anonymous. During session 4, trainees were informed that there would be a voluntary stakeholder engagement workshop at the final session. At the start of the workshop, voluntariness was re‐emphasised, and the facilitators would be taking handwritten notes, but any quotes would not be attributed to any individuals. As an evaluation, we did not have approval to collect any demographics.

### 2.10 Data Analysis

Quantitative analysis of the TARS results for each session and the overall training was conducted by generating descriptive statistics (frequencies, interquartile ranges and median averages [26]). TARS‐1 items were summed to calculate overall acceptability scores (possible range 6–36). TARS‐2 items were summed to calculate overall perceived impact scores (possible range 0–27). Overall TARS scores were calculated by summing the responses to all 15 questions (possible range 6–63).

The open‐ended comments, the facilitator notes and flip‐chart notes from the engagement workshop were analysed using content analysis [27], a qualitative method that can group open‐ended comments into categories that represent similar meanings and identify trends in the data by quantifying specific words or themes. A.G. facilitated analysis workshops with A.B., A.M., R.J. and S.M., which included guided reflexivity, and together they analysed the data and co‐constructed the findings, with A.G. and K.L. checking all the work.

### 2.11 Patient and Public Involvement

A.G. is an experienced LER [28]. A.G. modified the training content and also oversaw the delivery of the training and evaluation project. The topic guide for the workshop was co‐designed by A.G., A.B., A.M., R.J. and S.M. They had previously received training in content analysis from A.G. and analysed the data together.

## 3 Results/Findings

### 3.1 Trainees

In total, all 22 ‘involvement’ members attended the training. Attendance ranged from 13 to 22 trainees (mean 17.38), summarised in Table 3. For the engagement workshop, there were 18 attendees who took part.

**Table 3:** Attendance figures.

Session	In‐person	Via teams	Total
Intro	12	1	13
Reviews	15	1	16
Qual	12	2	14
Quant	15	2	17
Appraisal	14	3	17
Collab	16	4	20
H/Econ	18	2	20
Final	20	2	22

### 3.2 Quantitative Results

TARS‐1 has a possible score range of 1–6, and scores are detailed in Table 4. General appropriateness (Q1) of the sessions ranged 5–6 (moderately to strongly agree), with the quantitative session as a slight outlier (score 4, slightly agree). The effectiveness (Q2) of the sessions ranged 5–6. Trainees ‘strongly agreed’ that each session would not cause harm (Q3), with overall training scoring 5. The general approach (Q4) of sessions ranged 5–6. Trainees ‘strongly agreed’ that each session was consistent with common sense (Q5), with the introduction only slightly lower at a median of 5.5. Perceived approval (Q6) typically ranged 5–6, with the quantitative session as a slight outlier (score 4). Overall, these scores suggest that the training was acceptable to trainees, but that consideration could be given to slightly modifying the quantitative session.

**Table 4:** TARS‐1 Acceptability: *Range*, IQR and median average.

Session	*N*	Q1	Q2	Q3	Q4	Q5	Q6
Intro	12	*3–6* 1.5 **5**	*2–6* 1.5 **5**	*3–6* 1 **6**	*3–6* 2 **5**	*5–6* 1 **5.5**	*3–6* 1.5 **5**
Reviews	15	*3–5* 1 **5**	*3–6* 1 **5**	*3–6* 1 **6**	*3–6* 2 **6**	*3–6* 1 **6**	*3–6* 2 **5**
Qual	11	*4–6* 0 **6**	*5–6* 0 **6**	*4–6* 0 **6**	*5–6* 0 **6**	*5–6* 0 **6**	*5–6* 1 **6**
Quant	16	*2–6* 2 **4**	*3–6* 1 **6**	*3–6* 0 **6**	*3–6* 1 **6**	*3–6* 0.5 **6**	*2–6* 1 **4**
Appraisal	14	*1–6* 1 **5.5**	*1–6* 2 **5**	*1–6* 2 **6**	*1–6* 2 **5**	*1–6* 1 **6**	*1–6* 1 **5.5**
Collab	13	*3–6* 1 **6**	*3–6* 1 **6**	*3–6* 1 **6**	*3–6* 1 **6**	*3–6* 1 **6**	*3–6* 1 **6**
H/Econ	11	*4–6* 1 **6**	*4–6* 1 **6**	*6–6* 0 **6**	*5–6* 1 **6**	*5–6* 0 **6**	*4–6* 1 **5**
Course	17	*4–6* 1 **5**	*4–6* 1.5 **5**	*4–6* 1.5 **5**	*4–6* 1 **5**	*4–6* 1 **6**	*4–6* 1.5 **5**

TARS‐2 has a possible score range of 0–3, and scores are detailed in Table 5. The health economics session improved understanding (Q7) ‘a great deal’ (score 3), and all the other sessions had a median score of ‘quite a lot’ (score 2). Regarding improved skills (Q8), most topics had a median of ‘quite a lot’, except for the quantitative session, which was rated ‘a little’ (score 1). For improved confidence (Q9), ‘quite a lot’ was the median score for qualitative, appraisal, collaboration and health economics, and ‘a little’ for the introduction, reviews and quantitative sessions. In terms of future use (Q10), trainees could see themselves using the learning on collaboration ‘a great deal’, with the introduction, qualitative, appraisal and health economics sessions scoring ‘quite a lot’, and the reviews and quantitative sessions receiving a median score of ‘a little’. This suggests that future training could consider targeting improving confidence and demonstrating future use of most learning.

**Table 5:** TARS‐2 Perceived impact: *Range*, IQR and median average.

Session	*n*	Q7	Q8	Q9	Q10	Q11	Q12	Q13	Q14	Q15
Intro	12	*1–3* 0 **2**	*1–3* 1 **2**	*0–2* 1 **1**	*1–3* 1 **2**	*2–3* 1 **3**	*1–3* 1 **2**	*2–3* 1 **3**	*2–3* 1 **2.5**	*2–3* 1 **2**
Reviews	15	*1–3* 1 **2**	*1–2* 0 **2**	*1–2* 0 **1**	*1–2* 1 **1**	*1–3* 1 **3**	*1–3* 1 **3**	*2–3* 1 **3**	*2–3* 1 **3**	*2–3* 1 **2**
Qual	11	*1–3* 1 **2**	*1–3* 2 **2**	*1–3* 2 **2**	*2–3* 1 **2**	*2–3* 0 **3**	*2–3* 1 **3**	*2–3* 1 **2**	*2–3* 1 **3**	*2–3* 0 **3**
Quant	16	*1–3* 0 **2**	*1–3* 1 **1**	*1–3* 0.5 **1**	*1–3* 1 **1**	*2–3* 0 **3**	*2–3* 1 **3**	*2–3* 0 **3**	*2–3* 0 **3**	*2–3* 0 **3**
Appraisal	14	*1–3* 1 **2**	*1–3* 1 **2**	*1–2* 1 **2**	*0–3* 1 **2**	*1–3* 1 **3**	*1–3* 1 **2**	*1–3* 1 **3**	*1–3* 0 **3**	*1–3* 1 **3**
Collab	13	*1–3* 1.5 **2**	*1–3* 2 **2**	*1–3* 1 **2**	*2–3* 1 **3**	*1–3* 0 **3**	*1–3* 1 **3**	*1–3* 1 **3**	*1–3* 1 **3**	*2–3* 1 **3**
H/Econ	11	*1–3* 1 **3**	*1–3* 1 **2**	*1–3* 0 **2**	*1–3* 1 **2**	*2–3* 0 **3**	*2–3* 0 **3**	*2–3* 1 **3**	*2–3* 0 **3**	*2–3* 0 **3**
Course	17	*1–3* 0 **2**	*1–3* 0.5 **2**	*2–3* 0 **2**	*2–3* 1 **2**	*2–3* 1 **3**	*2–3* 1 **3**	*2–3* 1 **3**	*2–3* 1 **3**	*2–3* 1 **3**

In terms of overall satisfaction (Q12), trainees were satisfied ‘a great deal’ (score 3) with reviews, qualitative, quantitative, collaboration and health economics and were satisfied ‘quite a lot’ (score 2) with the introduction and the appraisal sessions. Regarding session coverage (Q13), most sessions were perceived to meet objectives ‘a great deal’, except for the qualitative session (‘quite a lot’). The trainers were competent (Q11, all scored 3), related well (Q14, most scored 3, except introduction at 2.5) and motivating (Q15, most scored 3, except introduction and reviews at 2).

Table 6 provides the overall acceptability, perceived impact and combined TARS scores. The collaboration (median 60/63), health economics (59) and qualitative (58) scored very highly, with critical appraisal (55), quantitative (54), reviews (53) and introduction (51) still scoring reasonably highly. With the overall course scoring 54/63, this still suggests an acceptable and impactful training provision.

**Table 6:** TARS acceptability, impact and combined overall median averages.

Session	Acceptability *n*/36	Perceived impact *n*/27	Combined TARS score *n*/63
Intro	31.5	19.5	51
Reviews	33	20	53
Qual	36	22	58
Quant	34	20	54
Appraisal	33	22	55
Collab	36	24	60
H/Econ	35	24	59
Course	31	23	54

### 3.3 Qualitative Findings

Six overarching themes were constructed: valued learning format; valuing research knowledge; valued the centring of lived experience; gaps in training provision; more lived experience research focussed; and consider further support of LERs (summarised in Table 7). Quotations are reported verbatim.

**Table 7:** Coding framework.

**Qualitative findings:**
**Valued learning format**
Group discussions (*n* = 11); more discussion (*n* = 3); larger groups (*n* = 1)
Q&A (*n* = 4); more Q&A (*n* = 2)
More case examples (*n* = 3)
Valued video (*n* = 2); more videos (*n* = 3)
Practical activities (*n* = 8)
Preparatory materials (*n* = 6)
Take‐home tasks (*n* = 3)
Further reading (*n* = 3)
Jargon buster (*n* = 2)
**Valued research knowledge**
Theoretical/conceptual learning (*n* = 11)
Methodological understanding (*n* = 5)
Expanding knowledge (*n* = 2)
Examples: Systematic review (*n* = 4); types of involvement (*n* = 3); quantitative (*n* = 1)
Further training topics (*n* = 2)
**Valued centring of lived experience**
LER facilitation (*n* = 10)
Case studies (*n* = 3)
The specific training slots (*n* = 11)
Demonstrating lived experience (*n* = 3)
Validating lived experience (*n* = 2)
**Gaps in training provision**
Application of new learning (*n* = 6)
Demonstrating influence (*n* = 5)
Relevance gap (*n* = 4)
Unaddressed tensions (*n* = 5)
Application to new learning (*n* = 3)
**More lived experience research focussed**
Focus throughout (*n* = 11)
Reflecting on experiential knowledge (*n* = 7)
Further tailored training (*n* = 2)
Further grounding in lived experience research (*n* = 4)
Further reading (*n* = 3)
**Consider further support of LERs**
Support considerations (*n* = 2)
Support within role (*n* = 2)
Peer support (*n* = 2)
Organisational support structures (*n* = 1)

#### 3.3.1 Valued Learning Format

Several trainees particularly valued the group discussions (*n* = 11 occurrences) and the opportunity for questions (*n* = 4 occurrences). There were a few requests for even more discussion (*n* = 1 for the review and *n* = 2 for appraisal), more Q&A (*n* = 2 for the review and collaboration sessions), one request for larger group discussions (for the introduction session), and three for more case examples (for the introduction, quantitative and collaboration sessions). There were three mentions of wanting video examples (for the introduction, review and appraisal sessions), with two further appreciations of the ‘Hidden’ video case study.

Some trainees particularly valued practical activities (*n* = 8 occurrences), such as the mind‐map (*‘Helpful self‐reflection on my own experience/knowledge’*), developing questions (‘*I liked trying to establish what questions were appropriate for the different research methods’*), a topic guide (‘*The process of developing a topic guide together—it was really effective at demonstrating method’*) and other skills (‘*Putting into practice methods such as critical appraisal skills’*), and one person noted when activities were absent (‘*Needs some creative activities’* [session 1]).

There were six requests for preparatory materials to be provided for sessions, including the slides and any research papers to be discussed. There were also three requests for activities framed as *‘take away’*, *‘home task’* or *‘practice homework’*. Moreover, there was a request for further reading material, with a clear rationale:

*‘Reading material for those who don't integrate information the first time around and need more time processing.’*

Another trainee requested *‘easy reads’* and another *‘Less technical papers to be analysed & shorter’*. Finally, there were two requests for a *‘jargon buster’* for *‘clarification of terminology’* and *‘breaking down language, as some phrases were used that I didn't understand and felt there wasn't time to ask.’* This suggests more could be done to make the training more accessible.

#### 3.3.2 Valuing Research Knowledge

Trainees named aspects of theoretical (e.g., *‘Good theory grounding’*) or conceptual learning (e.g., *‘I learnt a lot of new terms/concepts of study design’*) that were particularly helpful to them (*n* = 11 occurrences). Some particularly valued improved methodological *‘understanding’* (*n* = 5), and ‘*expanding*’ (*n* = 1) or ‘*broadening*’ (*n* = 1) knowledge.

Trainees particularly valued the systematic review process (*n* = 4), types of ‘involvement’ (*n* = 3) and different quantitative research designs (*n* = 1). Two people wanted further training on interviews and analysis.

#### 3.3.3 Valued the Centring of Lived Experience

LER facilitation was noted and appreciated for the introduction (*n* = 1), the qualitative (*n* = 2) and the collaboration (*n* = 3) sessions. It was noted as absent, but believed to be required, in the systematic review session (*n* = 1; *‘Needed E by E input’*) and the quantitative session (*n* = 2; *‘Needed lived experience presenter’*).

Three trainees particularly valued the lived experience research examples, including the qualitative session (*n* = 1; *‘Really good to hear about a lived experience PhD study!’*) and the collaboration session (*n* = 2; e.g., *‘Hidden LE project, really brought collaboration alive.’*). One trainee intimated that this would also be a helpful addition to the review session (‘*Would be good to use a lived experience example?’*), and there was a workshop comment that *‘more examples’* throughout the training course would be helpful.

Trainees valued the lived experience‐specific slots, including in the introduction (*n* = 1; *‘the Expert‐by‐Experience bit’*), the emotions in qualitative research (*n* = 1; *‘impacts on service users interviewing/analysing’*) and particularly the collaboration session (*n* = 4; e.g., *‘was good as made SU/carer involvement real’*). Five trainees particularly valued the mind‐map exercise (e.g., ‘*Useful way to reflect on LE/expertise/knowledge’*).

One trainee commented that, overall, they *‘really appreciated seeing a lived experience researcher in action’*. The value of all this was summed up by one workshop attendee: *‘Shows what we can achieve’*. Moreover, one trainee expressed the value as *‘helpful to see how lived experience shapes this kind of research’*. For another, it grounded them in their reason for being there:

*‘Lived experiences being a driving force for research reminded me of my own decision to be part of [the research programme]’*.

One trainee experienced the collaboration session as ‘*validating’*, another summed up their experience of the whole training as:

‘*I feel really valued and validated as an expert by experience—thank you!’*

Thus, LER facilitation, lived experience case examples and training content were valued aspects of the training.

#### 3.3.4 Gaps in Training Provision

Trainees identified five areas in which there were gaps in the training provision.

First, there were gaps related to the application of new learning. Six workshop stakeholders identified a gap in terms of not knowing how learning is going to be applied in the research programme. This was particularly related to the aims (*‘Not clear what the aims of the wider programme are, not sure how knowledge will be used in practice.’*) and the expectations (*‘Don't know the expectations on future use’*) of the programme, and the fact that people had not yet started in their roles *(‘Not really started yet, so difficult to assess application.’*).

Second, there were also gaps in demonstrating how lived experience involvement can influence research. For example, in relation to reviews, some (*n* = 3) wanted more content on lived experience involvement, its benefits, and how to influence reviews. For example, one trainee questioned:

*‘What's the benefit of involvement? How can we influence/be involved?’*

And another asked:

*‘What would these reviews lose without service user involvement? Unclear’*.

Two trainees raised similar issues about the quantitative session, for example:

*‘Need to bridge the gap between the research and the service user, don't see how we can be involved.’*

This suggests that, for these unique roles, training needs to show how people can concretely influence different research designs through lived experience involvement.

Third, for some sessions, there was also a perceived relevance gap. In relation to the quantitative session, there was a question of relevance (*n* = 1; *‘Relevance of these methods to service users?’*) and a perceived sense of distance (*n* = 1; *‘Some of it seems quite distant/difficult from lived experience.’*). One trainee also questioned the relevance of the review session:

*‘How does this session help service users in research?’*

Similarly, one trainee felt health economics would be inapplicable to them:

*‘When would we use?—not likely to be in a position to “role out” results.’*

This suggests that the training needs to help trainees see the relevance of learning for LERs.

Fourth, there was a largely unaddressed tension which was experienced by some between new learning and lived experience. In relation to reviews (*n* = 3), it was commented:

*‘How can our knowledge shape this kind of research, when it is so dismissive of our knowledge?’*

Similarly, in relation to the quantitative session:

*‘The pyramid of evidence bit was offputting! Language of bias difficult. Feels invalidating.’*

This issue was partly addressed in the final session, and it was commented on in the workshop:

*‘Helpful thinking that LE working fits with qual, tensions with quant.’*

This suggests that training could address the tensions LERs feel with quantitative research, which comes from a different research paradigm, with a different understanding of knowledge.

Finally, there was a key gap in terms of the training not sufficiently helping trainees to concretely apply their lived experience to their new learning, for example:

*‘It hasn't helped apply LE to research, a limitation.’*

There was thus some expectation that training should help people apply their experiential knowledge to their learning. The issue was only briefly addressed in the final training slot, which consolidated learning for two trainees, for example:

*‘This is where it all came together, would be good at the start’*

This suggests that training of LERs should focus on the application of lived experience to new learning throughout the training course.

#### 3.3.5 More Lived Experience Research Focussed

In different respects, 13 trainees wanted the training to be more lived experience research focussed. Some trainees wanted more of a focus on that right from the start of the training (e.g., *‘Lived experience right from the beginning’*) and then interwoven throughout the training (e.g., ‘*LE grounding session right at the start, then weave into research process etc.’*). In terms of specific methods, one trainee wanted the review session more tailored to lived experience:

*‘Different kinds of reviews/limitations from lived experience perspectives.’*

Another wanted a new, tailored session on ethics:

*‘Would like more on ethics/ethical approval in a separate session from a service user perspective.’*

This suggests that each session could be further tailored to LERs and that more content could be valuable.

Seven trainees found it particularly helpful to reflect on ‘experiential knowledge’. One person questioned whether this should come at the beginning:

*‘Helpful to consider the different ‘knowledge positions’ (SU, carer, public), perhaps this could be in the intro?’*

Another wanted more on the topic:

*‘More on experiential knowledge—and what is ‘lived experience’? Origins/development of ideas.’*

Furthermore, another suggested:

*‘A whole session on LER, knowledge issues, etc. would be great, at the beginning, and then applied throughout.’*

Two trainees found it helpful to reflect on the characteristics and limits of experiential knowledge. One person wanted more on the research paradigm tensions identified (*‘Helpful thinking that LE working fits with qual, tensions with quant—would like more on that.’)*. Another person, overall, wanted *‘More grounding in LE theory/practice’.* Two trainees requested a ‘*lived experience research reading list’*, and another wanted direction as to how to find survivor research.

#### 3.3.6 Consider Further Support of LERs

Nine trainees valued the fact that some of the sessions began to address some of the challenges LERs face in research, but they wanted more emphasis on support. Two trainees wanted training to address further such issues:

*‘How to focus back on hope and good after difficult LE interviews’.*

Another commented:

*‘Difficult to share LE, needs addressing more’*.

Some trainees wanted more support within the role (e.g., *‘Training on how to be mentored, keep good motivations, and share correct advice and support’.*). One person wanted *‘more on support for managing distress’.* Two people wanted information on peer support and its role. One person asked:

*‘How to ensure research meetings do not turn into therapy sessions?’*

There was also recognition of the importance and limitations of current organisational thinking on this matter:

*‘Importance of support structures came through, think organisations need to put more thought into this, particularly for supporting people with SMI.’*

This suggests that training should further consider the ongoing, unique support needs of LERs within research organisations.

## 4 Discussion

The findings add to the evidence on the difficulty of integrating lived experience within traditionally dominant research paradigms [29] in the context of research methods training for novice LERs. At times, there was a perceived training relevance gap, and future training should aim to show LERs how they can meaningfully *influence* the design and conduct of systematic reviews [30] and various quantitative study designs [31], and how to *apply* their experiential knowledge [29, 32, 33, 34]. LERs also experienced a tension between their lived experience and quantitative methods. Traditional researchers might not recognise this tension but may reinforce taken‐for‐granted epistemic hierarchies [35], consciously or unconsciously leading to the devaluation of experiential knowledge and expertise as epistemic injustice [36]. This knowledge/power dynamic may also work against co‐production approaches, whereby different forms of knowledge and expertise are to be valued equally [37]. All this suggests that the ‘standpoint’ from which the training is delivered is important to scrutinise.

Based on these findings, consideration should be given to delivering the whole training explicitly from the standpoint of lived experience, as informed by ‘standpoint theory’ [38]. This would require much more than LER co‐facilitation and the use of LER case studies throughout, but it would aim for established LERs to present lived experience research as a discipline, methodology and research paradigm, from an epistemic standpoint of ‘knowers of distress’ (or reflexive lived experience) [39]. Such training could also adopt a ‘critical pedagogy’ [40, 41], as opposed to the traditional pedagogy [17] that underpinned the original training, which would be more in tune with the emancipatory aims, activistic stance and ethical conduct [42, 43] of much of lived experience research.

The findings also challenge the normalisation stance underpinning the design of the original training, treating the course like any other university course and trainees as any other student. LERs are in unique roles that require tailored forms of training, help and support [44]. Traditional researchers may reinforce academic norms and lack an empathic understanding of the issues LERs face around ‘dual identity’ [44] and ‘emotional labour’ in research [45]. More consideration should be given to providing tailored mentorship, supervision, peer support and reflective practice [46]. However, as LERs have argued, organisational structures need to be put in place to support these roles [47].

### 4.1 Strengths and Limitations

A key strength of the training was the involvement of a LER in adapting the training content. Furthermore, the LER delivered significant aspects of the training. Whilst the work did not require formal ethical approval, a strength was that it was conducted to high ethical standards.

A strength of the training evaluation was the use of the TARS, and whilst there is a danger of constraining trainees' self‐assessment of learning, reducing complex learning to a rating scale, an approach which might stand in tension with the emancipatory aims of much of lived experience research and training [34], the open‐ended questions were particularly important to capture more nuanced data.

Limitations of the evaluation are that the sample size is small and consists of a single training cohort. A key limitation of the TARS is that it does not address the long‐term acceptability and actual impact in terms of applying learning in research/involvement practice, but only captures responses immediately after the training sessions. A further limitation was the lack of demographic data for the trainees.

The addition of an engagement workshop to further explore how the training could be adapted for LERs is a further strength of this study. Another was the addition of the LERs to co‐design the topic guide for the workshop and analyse the findings. A limitation to acknowledge is that A.G. and K.L. co‐facilitated the engagement workshop, when they themselves had delivered a significant proportion of the training. The possible effects of this were mitigated by workshop attendees writing down independently their comments/reflections on flip‐chart paper from small‐group discussions.

### 4.2 Future Research

The long‐term impact of training could be explored via a follow‐up study with ethical approval to collect participant demographics and explore experiences and impacts of training. Future work could incorporate case vignettes to capture learning in action. A bespoke training package delivered by established LERs for novice LERs will be developed and then formally evaluated.

## 5 Concluding Recommendations

- We recommend mixed‐methods research training for PPI contributors, to include LER co‐facilitators and using LER case studies and readings.
- Such training should help PPI contributors to apply their experiential knowledge to learning and to meaningfully influence research.
- We also recommend developing a bespoke training package by LERs for novice LERs, providing a grounding in lived experience research as a methodology/discipline/research paradigm.
- The wider support needs of LERs need further consideration.

## 6 A Lived Experience Commentary by C.B.

I have lived experience of poor mental health, and I was an attendee of this training programme. I am currently a service user representative and volunteer lived experience researcher for M‐RIC.

Overall, I think this is an excellent evaluation of the training. It's positive that our voices have been captured and used within the evaluation process.

This paper highlights the very important role of LER facilitators, when working with lived experience attendees, and how this positively impacts their learning outcomes. I feel the lived experience facilitation provided a mutual level of understanding of some of the challenges and hurdles faced by people with lived experience entering involvement in research. It also created what felt like a safe space for discussion and disclosure. Furthermore, the facilitation helped to engage the group and motivated us to talk more and ask questions without feeling judged.

The paper also suggests that the attendees strongly agreed that the ‘lived experience element’ should be present throughout the training programme, and this is something to consider when developing the training to deliver to future cohorts with lived experience. It made me feel valued as a person with lived experience and made me feel that lived experience was important and considered in a research context.

I agree with all the recommendations, and I believe that by using these recommendations in the future delivery of the training, it will help attendees to apply their own lived experience to research, in a way that supports their role.

## Author Contributions

**Andrew C. Grundy:** conceptualisation, methodology, investigation, analysis, writing – original draft, writing – review and editing. **Anam Bhutta:** analysis, writing – review and editing. **Ashgan Mahyoub:** analysis, writing – review and editing. **Rebecca Jenkins:** analysis, writing – review and editing. **Sadia Mir:** analysis, writing – review and editing. **Jahanara Miah:** funding acquisition, writing – review and editing. **Gail Faragher:** funding acquisition, writing – review and editing. **Karina Lovell:** funding acquisition, investigation, writing – review and editing, supervision.

## Disclosure

The views expressed in this publication are those of the authors and not necessarily those of the NIHR or the Department of Health and Social Care.

## Conflicts of Interest

The authors declare no conflicts of interest.

## Supporting information


**Supplementary Material:** TRAINING ACCEPTABILITY RATING SCALE (TARS) – adapted.

## Data Availability

The data that support the findings of this study are available from the corresponding author upon reasonable request.
