# Supplementary material for: Evaluation of a ‘Research Methods’ Training Course for Novice Lived Experience Researchers
Source: Health Expect. 2025 Aug 28;28(5):e70362. doi: 10.1111/hex.70362 (PMC12392132; doi:10.1111/hex.70362)
Supplement: Supplementary file 1 — Supplementary Material: TRAINING ACCEPTABILITY RATING SCALE (TARS) – adapted. [file HEX-28-e70362-s001.docx]

**TRAINING ACCEPTABILITY RATING SCALE (TARS) - adapted**

Training Course: .............................................................. Dates: ..................................

Instructions: Based on the training that you have just completed, please rate your agreement with the following statements on this scale:

1. = strongly disagree
2. = moderately disagree
3. = slightly disagree
4. = slightly agree
5. = moderately agree
6. = strongly agree

The first six statements concern the appropriateness or acceptability of the content of the training.

|  |  | CIRCLE YOUR LEVEL OF AGREEMENT | | | | | |
| --- | --- | --- | --- | --- | --- | --- | --- |
| 1 | **General acceptability:** This approach to training would be appropriate for a variety of service users. | 1 | 2 | 3 | 4 | 5 | 6 |
| 2 | **Effectiveness:** The training will probably be beneficial for others who receive it. | 1 | 2 | 3 | 4 | 5 | 6 |
| 3 | **Negative side-effects:**  The training will probably **not** cause harm to service users. | 1 | 2 | 3 | 4 | 5 | 6 |
| 4 | **Inappropriateness:** Most service users would accept that this training encouraged an appropriate approach. | 1 | 2 | 3 | 4 | 5 | 6 |
| 5 | **Consistency:** The training was consistent with common sense and good practice. | 1 | 2 | 3 | 4 | 5 | 6 |
| 6 | **Social validity:** Most service users would approve of the training (e.g. would recommend it to others). | 1 | 2 | 3 | 4 | 5 | 6 |

The next 12 questions focus on your impressions of the teaching process and outcomes (i.e. how competently you think the training was conducted, and whether it was helpful or not). For each question, please circle the statement that best expresses your opinion.

PLEASE CIRCLE ONE ANSWER:

| 7 | **Did the training improve your understanding?** | | | |
| --- | --- | --- | --- | --- |
|  | Not at all | A little | Quite a lot | A great deal |
| 8 | **Did the training help you to develop skills?** | | | |
|  | Not at all | A little | Quite a lot | A great deal |
| 9 | **Has the training made you more confident?** | | | |
|  | Not at all | A little | Quite a lot | A great deal |
| 10 | **Do you expect to make use of what you learnt in the training?** | | | |
|  | Not at all | A little | Quite a lot | A great deal |
| 11 | **How competent were those who led the training?** | | | |
|  | Not at all | A little | Quite a lot | A great deal |
| 12 | **In an overall, general sense, how satisfied are you with the training?** | | | |
|  | Not at all | A little | Quite a lot | A great deal |
| 13 | **Did the training cover the topics it set out to cover?** | | | |
|  | Not at all | A little | Quite a lot | A great deal |
| 14 | **Did those who led the training sessions relate to the group effectively?** (e.g. made you feel comfortable and understood) | | | |
|  | Not at all | A little | Quite a lot | A great deal |
| 15 | **Were the leaders motivating?** (e.g. energetic, attentive and creative) | | | |
|  | Not at all | A little | Quite a lot | A great deal |
| 16 | **What was the most helpful part of the training for you, personally?** | | | |
|  |  | | | |
| 17 | **What change, if any, would you recommend?** – particularly if you have any recommendations for better tailoring the session to the needs of service users (e.g. to the content or teaching) | | | |
|  |  | | | |
| 18 | **Please make any other comments that you would like to offer in relation to this Research Methods session for *Service Users*.** | | | |
|  |  | | | |
